# Do artificial neural networks provide improved volatility forecasts: Evidence from Asian markets

**DOI:** 10.1007/s12197-023-09629-8

**Published:** 2023-05-16

**Authors:** Mehmet Sahiner, David G. McMillan, Dimos Kambouroudis

**Affiliations:** Division of Accounting and Finance, University of Stirling, Stirling, FK9 4LA UK

**Keywords:** Volatility, Forecasting, Neural Networks, Machine Learning, VaR, ES, C22, C58, C63, G12, G17

## Abstract

This paper enters the ongoing volatility forecasting debate by examining the ability of a wide range of Machine Learning methods (ML), and specifically Artificial Neural Network (ANN) models. The ANN models are compared against traditional econometric models for ten Asian markets using daily data for the time period from 12 September 1994 to 05 March 2018. The empirical results indicate that ML algorithms, across the range of countries, can better approximate dependencies compared to traditional benchmark models. Notably, the predictive performance of such deep learning models is superior perhaps due to its ability in capturing long-range dependencies. For example, the Neuro Fuzzy models of ANFIS and CANFIS, which outperform the EGARCH model, are more flexible in modelling both asymmetry and long memory properties. This offers new insights for Asian markets. In addition to standard statistics forecast metrics, we also consider risk management measures including the value-at-risk (VaR) average failure rate, the Kupiec LR test, the Christoffersen independence test, the expected shortfall (ES) and the dynamic quantile test. The study concludes that ML algorithms provide improving volatility forecasts in the stock markets of Asia and suggest that this may be a fruitful approach for risk management.

## Introduction

The problem of forecasting stock market volatility remains a core issue in the empirical finance literature. While the impetus to earlier work began with the stock market crash of October 1987 (known as Black Monday), where twenty-three major world markets experienced substantial single day collapses,^1^ repeated market events serve to highlight the importance of understanding volatility. Latterly, this includes the global financial crisis (GFC) that began in 2007, where the S&P 500 saw a weekly drop of more than 20%, and the COVID-19 pandemic in March 2020, which led to a dramatic fall in global equity markets. The DJIA index slumped more than 26% in four trading days, while the price of WTI crude oil fell into negative territory for the first time in recorded history. Global stock markets lost over US$16 trillion within 52 days. As history indicates, wide swings in stock markets lead to greater uncertainties that can be followed by the anticipation of a potential financial crisis. Thus, interest in modelling and forecasting financial markets has grown over the years with a desire to improve understanding of crises, tail events, and systematic risk.

The first general approach applied to this task within the academic literature is the genre of GARCH models (Engle, 1982; Bollerslev, 1986), while from a practitioner’s viewpoint the RiskMetrics variance model (also known as Exponential Smoothing) was introduced by JP Morgan in 1989. Subsequently, the volatility index (VIX), based on S&P 500 index options, was developed by the Chicago Board Options Exchange (CBOE) to measure stock market expectations in 1993. The VIX index is often referred to as a fear gauge by market participants, while similar indexes have been developed for a range of markets.

These models, and their extensions, receive notable attention from both financial academics and practitioners with a large amount of related published work. Nevertheless, combined with the characteristic constraints on historical volatility models and the growing technological transformation of financial markets, this suggests that new technologies might be better placed to provide improved volatility forecasts. Machine learning models (ML), based on Artificial Intelligence (AI) technology, have significantly improved in recent years and financial markets provide a fertile ground to examine the accuracy of AI-based volatility models against those traditionally considered.

There is no doubt that with the advent of the digital computer, stock market prediction has since moved into the technological realm. Moreover, the gains in computational speed have become increasingly important for banks, hedge funds and retail investors who are required to make investment decisions both within a short period of time and with large and constantly updating information sets. As news can now be processed quickly, there is a large increase in the volume of transactions, which generate further volatility. Thus, to overcome such restraints and effectively address today’s noisy, fast-paced and non-linear markets, AI techniques are proposed. Brav and Heaton (2002) argue that traditional market theories and methods may become incompatible with, and thus unable to model, the sophistication of modern financial analysis. Therefore, recent improvements in information technologies and the noted success of machine learning in pattern recognition given their flexibility and feasibility motivates researchers to use AI algorithms in stock price prediction (Bebarta et al., 2012; Heaton et al., 2017). One of the most successful examples of AI applications in financial markets is the performance of Medallion Fund with an average return of 66% over the last two decades (Kamalov, 2020). As these models are capable of learning non-linear patterns and functions, they are also demonstrated to be universal function approximators (Hornik et al., 1989; Kosko, 1994).

The present paper aims to contribute to the finance literature by improving the volatility forecasts of Asian stock markets using sophisticated neural network and machine learning techniques, against traditional benchmark econometric models, using both standard and economic-based evaluation measures. In doing so, the benchmark indices of ten emerging and developed Asian stock markets with the data running from 12 September 1994 to 05 March 2018 are utilised. Although a broad number of studies investigate stock market volatility using ML methods, there is a limited number that examine Asian markets, particularly emerging ones. Asian economies are blossoming in the last few decades by contributing almost 30% to the global economic output and making up over 40% of the world population (Jordan et al., 2017). In recent years, some researchers separately investigate volatility in major Asian markets, including Chen and Hu (2022) for China, Shaik and Sejpal (2020) for India, and Harahap et al. (2020) for Japan. However, the financial markets of emerging countries such as Indonesia, Thailand, Malaysia, and the Philippines, which together constitute 66% of the market capitalization of the ASEAN economies as of 2016 (Ganbold, 2021), tend to be ignored in volatility exercises. Furthermore, Asian economies, particularly emerging ones, differ from western and other developed economies in terms of cultural, financial, and institutional characteristics, which causes variation in forecast accuracy (Chen et al., 2010; Dovern et al., 2015; Jordan et al., 2017). In addition, the highly volatile behavior of these markets has the potential to impact regional and global stock markets through both the ‘leverage effect’ and idiosyncratic risk factors (Atanasov, 2018; Bouri et al., 2020). This further indicates the importance of generating more accurate and comprehensive forecasts for these markets.

Overall, this study aims to fill the practical gap on the optimal forecast model for Asian stock markets by evaluating several ANN (artificial neural network) models based on static, dynamic and supervised learning techniques and compare them with traditional methodologies, including GARCH and EGARCH models. To the best of our knowledge, only a limited number of studies compare standard ANN, Neuro-Fuzzy, and deep learning models within a wide range of emerging and developed markets. In contrast to previous work, this paper adopts and builds advanced neural network architectures for each selected model with improved learning rules and optimized hyperparameters. Moreover, not only do we conduct a comprehensive comparison between traditional forecasting methods and ANN models, but we also examine the economic implications of these models by assessing measures relevant for risk management practice. In doing so, this paper presents results of importance to both academics and market participants including investors and regulators.

The content of the paper is organized as follows. In Section 2 we briefly provide a review of the existing literature. In Section 3 we discuss the methodology, followed by Section 4 where we provide the data. In Section 5 we present the results and end the paper with concluding remarks in Section 6.

## Literature review

Volatility forecasting is a prevalent topic that has attracted scholars over the years. Engle (1982) addresses the volatility estimation problem in developing the ARCH model, which is considered one of the most significant developments in the empirical financial literature. It is followed by the GARCH model of Bollerslev (1986) and subsequently, a number of GARCH extensions. The volatility literature also saw the development of alternative approaches, including, linear regression models, hybrid models, the support vector regression, fuzzy logic, genetic algorithms and artificial neural networks.

One of the earliest studies on machine learning forecasting is Yoon and Swales (1991) who examine the stock market data of 58 widely followed companies in the Fortune 500. Their findings reveal that neural networks significantly improve stock price predictability compared to conventional methods. Donaldson and Kamstra (1996) improve the applicability of the ANN approach using time series data on four developed stock markets. They conduct out-of-sample forecasts and reveal that ANN is superior in terms of forecasting stock market volatility compared to traditional linear models given its flexibility with complex nonlinear dynamics. Furthermore, Ormoneit and Neuneier (1996) apply ANN models on the German DAX index by using minute data for the month of November 1994. They compare the Multilayer Perceptron method (MLP) with the Conditional Density Estimating Neural Network (CDENN) and report that improved predictions can be achieved by more complex architectures that target noise in the stock market. In light of this study, Kim and Lee (2004) propose the feature transformation method based on the Genetic Algorithm (GA) model and compare it with two conventional neural networks. The results indicate that the GA method improves prediction capability for financial market forecasting compared to the conventional ANNs. Altay and Satman (2005) implement ANN methods on the Istanbul Stock Exchange by using daily, weekly, and monthly data. They compare out-of-sample forecasting results with linear regression models and report that ANN is superior only for weekly forecast results, while underperforming for daily and monthly data. Cao et al. (2005) study ANN methods to predict firm-level stock prices that trade on the Shanghai stock exchange. They compare univariate and multivariate ANN models with linear models, with the results indicating superiority of the neural network models in predicting future price changes. In contrast, Mantri et al. (2014) investigate the two Indian benchmark indices (BSE SENSEX and NIFTY) from 1995 to 2008 by comparing GARCH, EGARCH, GJR-GARCH, IGARCH and ANN models. The authors report that the prediction ability of the ANN model offers no improvement over statistical forecast models.

A number of studies investigate the performance of a different class of ANN and hybrid models. Roh (2007) proposes a hybrid model between ANN and time series econometric models for the KOSPI Index, with forecast results supporting the accuracy of the hybrid model for volatility. Unlike Roh (2007), Guresen et al. (2011) analyse daily NASDAQ returns but find that hybrid models are not as successful as standard ANN models. Kristjanpoller et al. (2014) propose ANN-GARCH hybrid models to predict three emerging Latin American stock markets and conclude that hybrid models improve prediction ability over conventional models. Further studies relating to hybrid models are undertaken by Rather et al. (2015), Mingyue et al. (2016), Kim and Won (2018), and Hao and Gao (2020).

Adebiyi et al. (2012) combine technical and fundamental analysis with ANN and provide results suggesting that the combination improves prediction, consistent with the findings of Yao et al. (1999) and later supported by Sezer et al. (2017). However, Namdari and Li (2018) report mixed results in terms of forecasting ability of an integrated ANN with fundamental and technical analysis models. Of note, the results show that the integrated model works well when the stocks have an upward trend.

Several researchers experiment with neuro-fuzzy and neuro-evolutionary methods in stock market forecasting exercises, which are believed to have a combination of advantages through ANN and fuzzy logic. Quah (2007) uses DJIA index data spanning from 1994 to 2005 to compare the applicability of MLP, ANFIS and GGAP-RBF models. Using several benchmark metrics, including generalize rate, recall rate, confusion metrics and appreciation, the study shows that ANFIS provides more accurate results while GGAP-RBF underperforms in all selected criteria. In a similar vein, Yang et al. (2012) find that a fuzzy reasoning system can be used to predict stock market trends. Li and Xiong (2005) argue that neural networks have limitations in dealing with qualitative information and suffer from ‘black box’ syndrome, proposing a neuro fuzzy inference system to overcome these drawbacks. The Shanghai stock market is chosen for prediction where they find the suggested fuzzy NN is superior to standard NN methods. Mandziuk and Jaruszewicz (2007) propose a novel neuro-evolutionary method to predict the change in the daily closing price on the DAX index. The results reveal that the proposed model produces high accuracy for the market in both directions. García et al. (2018) implement a hybrid neuro fuzzy model to predict one-day ahead direction of the DAX Index. They conclude that the integration of traditional indicators with ANN may enhance predictive accuracy of the model, although it may also generate noise in the prediction model. Further discussion is reported in D’Urso et al. (2013), Vlasenko et al. (2018) and Chandar (2019).

More recently, Luo et al. (2018) discuss that the predictive capabilities of deep learning models are lesser compared to other ANN algorithms. Although D’Amato et al. (2022) demonstrate the suitability and capability of deep learning models on the inherently complex and chaotic crypto market. Koo and Kim (2022) propose a new model by combining GARCH and LSTM models with a volume-upped (VU) distribution strategy. They conclude that the proposed model improves forecasting performance by 21.03% compared to standalone deep learning models. Similarly, Ahamed and Ravi (2021) investigate the shortcomings of a deep learning network by focusing on the optimization problem and address the issue by using swarm intelligence algorithms. Whereas Yang et al. (2020) reveal that the predictive power of genetic algorithms is better than the swarm optimization.

The above discussion demonstrates that the present state of the literature does not suggest a clear superiority either within the different ANN models, or over conventional forecasting methods. As Ravichandra and Thingom (2016) and Chopra and Sharma (2021) discuss, AI models do possess superior capabilities and the potential for more accurate volatility forecasts and thus, worthy of further research. This paper builds upon the research in the current literature comparing the volatility forecasting capabilities of ML models to traditional models and extends the literature by evaluating a wider set of ANNs and utilising risk management measures and so developing the economic implications.

## Empirical methodology

### Benchmark models

#### Naïve forecast

Naïve forecasts are the most basic and cost-effective forecasting models that provide a benchmark against more complex models. This technique is widely used in empirical finance, especially for time series that have difficult to predict patterns. Forecasts are calculated based on the last observed value. Hence, for time *t*, the value of observation at time *t* − 1 is considered the best forecast:

1 $${\hat{y}}_t={y}_{t-1}$$

Where *y*_*t-1*_ represents the volatility proxy (squared returns).

*The Moving Average Convergence Divergence Indicator (MACD)*

MACD is a technical indicator designed by Gerald Appel in the late 1970s to reveal changes in the strength, momentum, and trend of stock prices. The standard MACD is calculated by subtracting the 26-period Exponential Moving Average (EMA) from the 12-period EMA as:

2 $$MACD=12\ period\ EMA-26\ period\ EMA$$

3 $$Signal\ Line=9\ period\ EMA\ of\ the\ MACD$$

When applied to stock prices, the MACD produces trading signals. When MACD falls below the signal line, it is a bearish signal and indicates a sell. Conversely, when MACD rises above the signal line, this is a bullish signal and indicates a buy. With respect to volatility, this approach essentially captures up and down trends within the volatility proxy.

#### **GARCH family models**

The GARCH approach forms the baseline model for this study. While there are over 300 GARCH-type models (Hansen and Lunde, 2005), we consider two of the most widely used, the GARCH (Bollerslev, 1986) and the EGARCH (Nelson, 1991) models. As these models are well-known in the literature, we provide only a brief description. The return specification is given by:

4 $${r}_t=\mu +{\varepsilon}_t$$

where *r*_*t*_ is the return series, *μ* is the constant mean and *ε*_*t*_ = *h*_*t*_*z*_*t*_ refers to the return residuals, which contain the volatility signal, *h*_*t*_, and an *i.i.d.* residual term, *z*_*t*_, with 0 mean and 1 variance *(i.i.d.)*. The conditional variance specifications of the chosen models are as follow:

5 $$\textrm{GARCH}:\kern0.5em {h}_t^2={\alpha}_0+{\alpha}_1{\varepsilon}_{t-1}^2+\beta\ {\textrm{h}}_{t-1}^2$$

6 $$\textrm{EGARCH}:\kern0.5em \ln \left({h}_t^2\right)={a}_0+{\beta}_1\ln \left({h}_{t-1}^2\right)+{a}_1\left\{\left|\frac{\varepsilon_{t-1}}{h_{t-1}}\right|-\sqrt{\frac{2}{\pi }}\right\}-\gamma \frac{\varepsilon_{t-1}}{h_{t-1}}$$

where ${h}_t^2$ is the time-dependent conditional variance and *α*_0_, *a*_1_, *β* and *γ* are the parameters estimated using the maximum likelihood method.

### Artificial neural networks

Artificial Neural Networks (ANNs) are one of the most popular approaches in machine learning applications. ANNs are brain-inspired models which imitate the network of neurons in a biological brain so that the computer will be able to learn and make decisions in a human-like manner. There are several types of ANN models developed for specific applications, including for pattern recognition and financial market prediction. In this section, these network types will be introduced.

#### Multi-Layer Perceptron (MLP)

A multi-layer perceptron (MLP) is a feedforward (where the information moves forward from input to output nodes) artificial neural network and is one of the most known and used neural network architectures in financial applications according to Bishop (1995). The MLP model with one hidden layer is given as follows:

7 $${n}_{k,t}={w}_{k,0}+\sum_{i=1}^i{w}_{k,i}{x}_{i,t}$$

8 $${N}_{k,t}=L\left({n}_{k,t}\right)=\frac{1}{1+{e}^{-{n}_{k,t}}}$$

9 $${Y}_t={\lambda}_0+\sum_{k=1}^k{\lambda}_k{N}_{k,t}$$

where *i* shows the number of input data (*x*) and *k* represents the number of nodes (neurons). The activation (transfer) function is chosen as logistic sigmoid function due to its convenience and popularity which is represented by *L*(*n*_*k*, *t*_) and defined as ${~}^{1}\!\left/ \!{~}_{1+{e}^{-{n}_{k,t}}}\right.$.

The training process starts with the input vector *x*_*i*, *t*_, weight vector *w*_*k*, *i*_, and the coefficient variable *w*_*k*, 0_. Combining these input vectors with the squashing function log-sigmoid, forms the neuron *N*_*k*, *t*_, which then serves as an exogenous variable with the coefficient *λ*_*k*_ and the constant *λ*_0_ to forecast output *Y*_*t*_. This network architecture with the logarithmic sigmoid transfer function is one of the most popular methods used to forecast financial time series data (Emerson et al., 2019; Sermpinis et al., 2021).

#### Recurrent Neural Network (RNN)

A Recurrent Neural Network (RNN) is a class of artificial neural network that allows the process of sequential information. In the RNN architecture, previous outputs can be used as inputs while having hidden states. The main difference between basic feedforward networks and RNN is that RNNs can impact the process of future inputs. In other words, feedforward networks can only ‘remember’ things that they learnt during training, while RNNs can learn during training. In addition, they remember things learnt from prior input while generating output. As in the moving average model where the endogenous variable *Y* is a function of an exogenous variable *X* and an error term *ε*, likewise, nodes in the RNN are a function of input data and its previous value from *t* − 1. The equation of RNN is given as follows:

10 $${n}_{k,t}={w}_{k,0}+\sum_{i=1}^i{w}_{k,i}{x}_{i,t}+\sum_{k=1}^k{\varphi}_k{n}_{k,t-1}$$

11 $${N}_{k,t}=\frac{1}{1+{e}^{-{n}_{i,t}}}$$

12 $${Y}_t={\lambda}_0+\sum_{k=1}^k{\lambda}_k{N}_{k,t}$$

The advantages of RNNs, which include having short-term ‘memory’ and the ability to process sequential datasets, attract broad attention among financial researchers and various applications are conducted (Rather et al., 2015; Gao, 2016; Samarawickrama and Fernando, 2017; Pang et al., 2020). However, the difficulty of training and the requirement of additional connections are the major drawbacks for RNN architectures. RNNs are also prone to the problem of gradient vanishing, which is the phenomenon of difficulty in capturing long-term dependencies. It occurs when more layers using certain activation functions are added to the network, which causes the gradients of the loss function to approach zero, making the network hard to train. To overcome this issue, Hochreiter and Schmidhuber (1997) propose the Long Short-Term Memory (LSTM) networks. LSTMs are proficient in training long-term dependencies and improve transformation with additional gates and a cell state. The structure of LSTMs is slightly different from conventional RNNs where RNNs have standard neural network architecture with a feedback loop, LSTMs contain three memory gates namely input gate, output gate and forget gate as well as a cell. The purposes of these gates are:

- The input gate states which information to add to the memory (cell).
- The output gate specifies which information from the memory (cell) to use as output.
- The forget gate describes which information to remove from the memory (cell).

LSTMs are considered ‘state of the art’ systems in forecasting time series data, pattern recognition and sequence learning.

#### Modular Feedforward Networks (MFNs)

Modular Feedforward Networks (MFNs) are an extension of typical feedforward NN architectures that are designed to reduce complexity and enhance robustness. The issues of learning weights and slow convergence in standard NN designs, motivate researchers to study new designs to generate more efficient results.

The MFNs have several different networks that function independently and perform sub-tasks. These different networks do not interact with or signal each other during the computation process. They work independently towards achieving the output (see Tahmasebi and Hezarkhani, 2011).

#### Generalized Feedforward Networks (GFNs)

Generalized Feedforward Networks (GFNs) are a subclass of Multi-layer Perceptron (MLP) networks that enable connections to jump over one or more than one layer. The direct connections between two separate layers provide raw information for the output layer along with the usual connection via the hidden layer.

The most prominent feature of GFN is providing the capability to send linear connections if the underlying elements consist of a linear component. But, if the underlying elements require non-linear connectivity, then the jump function is not needed. Theoretically, MLP can provide solutions to every task that GFN architecture can overcome. However, in practice, GFNs offer more accurate and efficient solutions compared to standard MLP networks. The GFNs are applied in many areas, including time series forecasting, data processing, pattern recognition and complex engineering problems. For further information, see Arulampalam and Bouzerdoum (2003) and Celik and Kolhe (2013).

#### Radial Basis Function Networks (RBFNs)

Radial Basis Function Networks (RBFNs) are a three-layered feedforward network that use radial basis function as activation function. The architecture was developed by Broomhead and Lowe (1988) to increase speed and efficiency of Multi-Layer Perceptron Networks as well as reducing the parameterization difficulty. The standard RBFN process is given by McNelis (2005) as follows:

13 $${\mathit{\operatorname{Min}}}_{<\omega, \mu, \tau >}\sum_{t=0}^T{\left(\ {y}_{t-}{\hat{y}}_t\ \right)}^2$$

14 $${n}_t={w}_0+\sum_{i=1}^{i^{\ast }}{w}_i{x}_{i,t}$$

15 $${R}_{k,t}=\phi \left({n}_t;{\mu}_k\right)$$

16 $$=\frac{1}{\sqrt{2\pi {\sigma}_{n-{\mu}_k}}}\mathit{\exp}{\left(\frac{-\left[{n}_t-{\mu}_k\right]}{\sigma_{n-{\mu}_k}}\right)}^2$$

17 $${\hat{y}}_t={\lambda}_0+\sum_{k=1}^{k^{\ast }}{\lambda}_k{R}_{k,t}$$

where:

*x*: the set of input variables
*n*: the linear transformation of the input variables
*w*: weights.

The parameter *k*^∗^ shows the number of centres for the transformation function of radial basis *μ*_*k*_, *k* = 1, 2, …*k*^∗^ computes the error function generated by the separate centres, *μ*_*k*_, and obtains the *k*^∗^ separate radial basis function, *R*_*k*_. The vector *σ* is used to represent the width associated with each of the radial centre. These parameters are then used to estimate the output ${\hat{y}}_t$ with weights *λ* via the linear transformation, after which, the RBFN optimization occurs and includes determination of parameters *w*, *λ* with *k*^∗^ and *μ*.

#### Probabilistic Neural Networks (PNNs)

Probabilistic Neural Networks (PNNs) was developed by Specht (1990) to overcome the classification issue caused by the applications of directional prediction. The structure of PNNs is formed of four layers which are the input layer, the pattern layer, the summation layer, and the output layer.

The linear and adaptive linear prediction designs of PNNs are the most popular functions in forecasting exercises of time series. The main advantages of PNNs compared to MLPs are requiring less training time, providing more accuracy and being relatively less sensitive to outliers. The main disadvantage of the PNNs is the requirement of more memory space to store the model.

#### Adaptive Neuro-Fuzzy Inference System (ANFIS)

Adaptive Neuro-Fuzzy Inference System (ANFIS) is a subclass of ANNs introduced by Jang (1993). According to Yager and Zadeh (1994), the model is considered one of the most powerful hybrid models as it is based on two different estimators, namely Fuzzy Logic (FL) and ANN, which are designed to produce accurate and reliable results by justifying the noise and ambiguities in complex datasets. The ANFIS architecture is based on the Takagi-Sugeno inference system, which generates a real number as output. The structure of the model is similar to a MLP network with the difference in flow direction of signals between nodes and exclusion of weights. The simulation of the ANFIS model and the function of each layer is presented as follows:

- *Layer 1: Selection of input data and process of fuzzification*

In this step input parameters are chosen and the fuzzification is initialized by transforming crisp sets into fuzzy sets. This process is defined as follows:

18 $${O}_{1i}=\mu {A}_i\left({x}_1\right),\kern2.75em {O}_{2i}=\mu {B}_i\left({x}_2\right),\kern1.5em for\kern0.5em i=1,2$$

where *x*_1_ and *x*_2_ are input parameters, *A*_*i*_ and *B*_*i*_ are linguistic labels of input parameters, *O*_1*i*_ and *O*_2*i*_ are membership grades of fuzzy set *A*_*i*_ and *B*_*i*_.

- *Layer 2: Computation of firing strength*

This layer is also called as rule layer and the outcome of this layer is known as firing strength. The nodes in this layer are fixed and represented by Π. These nodes are responsible for receiving information from the previous layer and the output of these nodes is obtained by the following equation:

19 $${w}_i=\mu {A}_i\left({x}_1\right)\mu {B}_i\left({x}_2\right)\kern1.5em for\kern0.5em i=1,2$$

- *Layer 3: Normalization of firing strength*

Each node is fixed in the 3^rd^ layer and defined as Ν. The nodes in this layer receive signals from each node in the previous layer and calculate the normalized firing strength by the given rule:

20 $${\overline{w}}_i=\frac{w_i}{w_1+{w}_2}\kern4.5em for\kern0.5em i=1,2$$

- *Layer 4: Consequent Parameters*

The nodes in this layer are adaptive and process the information from 3^rd^ layer by a given rule as follows:

21 $${\overline{w}}_i{f}_i={\overline{w}}_i\left({p}_i{x}_1+{q}_i{x}_2+{r}_i\right)\kern3.25em for\kern0.5em i=1,2$$

where ${\overline{w}}_i$ is the normalized firing strength and *p*_*i*_, *q*_*i*_, *r*_*i*_ are the parameter(s) set that can be determined by the method of least squares.

- *Layer 5: Computation of overall output*

This layer is labeled as Σ and contains only a single node which calculates the overall ANFIS output by aggregating all the information received from 4^th^ layer:

22 $$y=\sum_i{\overline{w}}_i{f}_i=\frac{\sum_i{w}_i{f}_i}{\sum_i{w}_i}$$

The mathematical details of ANFIS training procedure can be obtained in the studies of Jang (1993), Jang et al. (1997), Nayak et al. (2004), and Tahmasebi and Hezarkhani (2011).

#### Co-Active Neuro-Fuzzy Inference System (CANFIS)

The Co-Active Neuro-Fuzzy Inference System (CANFIS) is an extended version of ANFIS architecture and was introduced by Jang et al. (1997). The main advantage of CANFIS is the ability to deal with any number of input-output datasets by incorporating the merits of both neural network (NN) and fuzzy inference system (FIS) (Mizutani and Jang, 1995; Aytek, 2009). The main distinctive elements of CANFIS are the fuzzy axon (a) which applies membership functions (all the information in fuzzy set) to the inputs and a modular network and (b) that applies functional rules to the inputs (Heydari and Talaee, 2011).

As in the ANFIS system, the CANFIS system is also based on Sageno function. The main contribution of CANFIS model is to provide multiple outputs, while the two biggest drawbacks of the system are (a) problem with dealing extreme values and (b) the requirement of a large dataset to train the model.

#### Forecast combination

To provide some overview of the ANN models, we consider a simple forecast combination approach. The combination of forecasts is generally considered a useful tool to improve performance of individual forecasts. The arithmetic average method can be used with various forecasting models, which provides robustness and accuracy to the overall results. This method is applied as follows:

23 $$ANN\ {Fc}_t=\left({f}_t^{NN1}+{f}_t^{NN2}+\dots +{f}_t^{NN m}\right)/m$$

where ANN Fc is the forecast combination, ${f}_t^{NN}$is the Neural Network forecast at time *t* and *m* is the number of forecasts.

### Neural network implementation

#### Hidden layers

The learning process of a neural network is performed with layers and where the hidden layer(s) plays a key role in connecting input and output layers. Theoretically, a single hidden layer with sufficient neurons is capable of approximating any continuous function. Practically, single or two hidden layers network is commonly applied and provides good performance (Thomas et al., 2017). Therefore, this study follows the maximum of two hidden layers approach for each NN model.

#### Epochs

The number of epochs is a hyperparameter that defines the number of times that the learning algorithm will work through the entire training dataset (Brownlee, 2018). The default number of 1000 epochs is used for training the data, but early stopping is applied if there is no improvement after 100 epochs to prevent overfitting (Prechelt, 2012).

#### Weights

Weights are the parameters in a neural network system that transforms input data within the network’s hidden layers. A weight decides how much influence the input will have on the output. Negative weights reduce the value of an output. The reproduction phase of the models is performed based on two modes of weight update, which are online weighting and batch weighting. In batch mode, changes to the weight matrix are accumulated over an entire presentation of the training data set, while online training updates the weight after the presentation of each vector comprising the training set.

#### Activation function

The activation function (also known as the transfer function) determines the output of a neural network by a given input or set of inputs. The use of the activation function is to limit the bounds of the output values that can ‘paralyze’ the network and prevent the training process. The activation functions can be divided into two groups of linear and non-linear activation functions. As Hsieh (1995) and Franses and Van Dijk (2000) state, the fact that financial markets are non-linear and exhibit memory, suggests that non-linear activation functions are more suitable for forecast tasks. While there are various types of non-linear transfer functions, this study adopts the tanh activation function as such:

24 $${y}_i(t)=f\left({x}_i(t),{w}_i\right)$$

where *y*_*i*_(*t*) is the output, *x*_*i*_(*t*) is the accumulation of input activity from other components and *w*_*i*_ is the weight, with:

25 $$\tanh (x)=\frac{2}{1+{e}^{-2x}}-1,\kern3.25em f\left({x}_i,{w}_{,}\right)=\left\{\begin{array}{c}\begin{array}{c}-1\kern0.75em {x}_i<-1\\ {}1\kern1.25em {x}_i>1\end{array}\\ {}{x}_i\kern1.25em else\end{array}\right\}$$

The tanh function is extensively used in time series forecasting as it delivers robust performance for feedforward neural networks, see Gomes et al. (2011), Zhang (2015) and Farzad et al. (2019).

#### Learning rule

The learning rule in a neural network is a mathematical method to improve ANN performance via helping a neural network to learn from the existing conditions. The Levenberg-Marquardt (LM) algorithm, used in this study, is designed to work specifically with loss functions. This method, developed separately by Levenberg (1944) and Marquardt (1963), provides a numerical solution to the problem of minimizing a non-linear function (Yu and Wilamowski, 2011). It is one of the fastest methods to train a network and has stable convergence, making it one of the more suitable higher-order adaptive algorithms for minimizing error functions.

### Forecast methodology and evaluation

The empirical models and out-of-sample forecasts used in this study are estimated and generated using a recursive window method. The choice of estimation window for the out-of-sample forecasts is an area of debate, as no effective solution is proposed for the optimal length. Thus, an adequately large window length is recommended, specifically when considering richly parameterized neural network models (Cerqueira et al., 2019). In this context, the full sample period is divided into two sub-samples, with the out-of-sample forecasts in the second period are obtained based on the parameters estimated in the first. Table 1 reports the sample sizes and out-of-sample forecasting periods for each index.

**Table 1:** Sample sizes and Out-of-sample forecasting period for daily return series in selected markets

Stock Market	Estimation Period	Estimation Size	Forecast Period	Forecast Size	Full Sample Size
NIKKEI	12/09/1994 - 8/11/2006	2874	8/14/2006 - 5/02/2018	2876	5750
STI	8/31/1999 - 12/30/2008	2344	12/31/2008 - 5/02/2018	2346	4690
HSI	1/10/1995 - 8/29/2006	2874	8/30/2006 - 5/03/2018	2877	5751
KLCI	1/10/1995 - 9/04/2006	2871	9/05/2006 - 4/30/2018	2869	5740
JCI	1/11/1995 - 8/25/2006	2847	8/28/2006 - 4/26/2018	2841	5688
SET	1/11/1995 - 8/24/2006	2855	8/25/2006 - 4/25/2018	2848	5703
SSE	1/10/1995 - 9/11/2006	2828	9/12/2006 - 5/03/2018	2829	5657
TAIEX	1/11/1995 - 3/23/2006	2997	3/24/2006 - 5/02/2018	2895	5892
KOSPI	1/10/1995 - 5/02/2006	2970	5/03/2006 - 5/02/2018	2973	5943
PSE	1/11/1995 - 7/17/2006	2875	7/18/2006 - 5/02/2018	2853	5728

A range of well-known forecast metrics are utilized for evaluation. This includes the mean absolute error (MAE), mean absolute percentage error (MAPE), mean squared error (MSE), root mean squared error (RMSE) and Quasi-Likelihood Loss Function (QLIKE):

26 $$MAE=\frac{1}{n}\sum_{t=1}^n\left|{\sigma}_t^2-{\hat{\sigma}}_t^2\right|$$

27 $$MAPE=\frac{1}{n}\sum_{t=1}^n\frac{\left|{\sigma}_t^2-{\hat{\sigma}}_t^2\right|}{\sigma_t^2}$$

28 $$MSE=\frac{1}{n}\sum_{t=1}^n{\left({\sigma}_t^2-{\hat{\sigma}}_t^2\right)}^2$$

29 $$RMSE=\sqrt{\frac{1}{n}\sum_{t=1}^n{\left({\sigma}_t^2-{\hat{\sigma}}_t^2\right)}^2}$$

30 $$QLIKE=\frac{1}{n}\sum_{t=1}^n\left(\log \left({\hat{\sigma}}_t^2\right)+\left(\frac{\sigma_t^2}{{\hat{\sigma}}_t^2}\right)\right)$$

In each case, *n* denotes the number of forecast data points, ${\sigma}_t^2$ is the true volatility series (Andersen and Bollerslev, 1998) which is obtained by the squared return series and ${\hat{\sigma}}_t^2$ is the forecasted conditional variance series at time *t*. Of note, Patton and Sheppard (2009), Patton (2011), and Conrad and Kleen (2018) argue that the MSE and QLIKE are more reliable in volatility forecasting.

#### Model confidence set test

Although the above evaluation metrics allow forecasts to be ranked, it is difficult to determine whether there are any statistically significant differences in the values. To draw such conclusions, the paper implements the Model Confidence Set (MCS) method of Hansen et al. (2011). This procedure follows a sequence of statistical tests that allows for production of a set of ‘superior’ models. The MCS eliminates the worst performing model sequentially based on the equal predictive ability (EPA) approach until the final MCS includes the optimal model(s) according to a given confidence level.

Formally, the procedure starts with the set of alternative candidate forecasting models, defined by *M*_0_ = 1, 2, …, *m*_0_. Then to evaluate the performances among selected forecasts, all loss differentials between models are calculated as follows:

31 $${d}_{ij,t}={L}_{i,t}-{L}_{j,t},\kern0.5em for\ all\ i,j\in {M}_0$$

where *d*_*ij*, *t*_ denotes the loss differential between the loss functions of the *i*^th^ model and *j*^th^ model at time *t*. For the given set of models the null hypothesis and alternative hypothesis of EPA are formulated as follow:

32 $${H}_{0,M}:E\left({d}_{ij,t}=0\right),\kern1.75em \forall i,j={M}_0$$

33 $${H}_{A,M}:E\left({d}_{ij,t}\ne 0\right),\kern1.5em for\ some\kern0.5em i,j={M}_0$$

The MCS sequential testing procedure starts by testing the null hypothesis of EPA at each stage using the given significance level and if it is rejected, the significantly inferior model is eliminated until the first non-rejection occurs. However, in order to decide whether the MCS would further reduce at any step, the null hypothesis of EPA in equation (32) must be estimated at each step of the process. To address this drawback, Hansen et al. (2011) propose the Range Statistic and Semi-quadratic statistic, defined as:

34 $${T}_R=\underset{i,j\in {M}_0}{\max}\frac{\left|{\overline{d}}_{i,j}\right|}{\sqrt{{\hat{\mathit{\operatorname{var}}}\Big(}{\overline{d}}_{i,j}\Big)}}\kern1.25em and\kern0.75em {T}_{SQ}=\kern0.5em {\sum}_{i,j\in {M}_0}\frac{{\left({\overline{d}}_{i,j}\right)}^2}{\hat{\mathit{\operatorname{var}}}\left({\overline{d}}_{i,j}\right)}\kern0.5em$$

where ${\overline{d}}_{i,j}$ denotes the mean value of *d*_*ij*, *t*_, given by ${\overline{d}}_{i,j}={~}^{1}\!\left/ \!{~}_{M}\right.\sum {d}_{ij,t}$.

### Risk management

#### Value at Risk (VaR) and Expected Shortfall (ES)

We also consider economic loss functions. Value at Risk (VaR) measures and quantifies the level of risk over a specific interval of time. Jorion (1996) defines VaR as the worst expected loss over a target horizon under normal market conditions at a given level of confidence. Due to its relative simplicity and ease of interpretation, it has become one of the most commonly used risk management metrics. However, VaR has several drawbacks including the issue that it does not measure any loss beyond the VaR level, which is also referred to as ‘tail risk’ (Alexander, 2009; Danielsson et al., 2016). To overcome this, Artzner et al. (1999) introduce Expected Shortfall (ES), which is also known as conditional Value at Risk (CVaR), average value at risk (AVaR), and expected tail loss (ETL). Expected Shortfall measures the conditional expectation of loss exceeding the Value at Risk level. Where VaR asks the question of “How bad can things get?”, ES asks “If things get bad, what is our expected loss?”. We evaluate the forecast models using both of these metrics. VaR is defined as:

35 $$VaR={\mu}_t+{\sigma}_tN\left(\alpha \right)$$

where *μ*_*t*_ is the mean of the logarithmic transformation of daily return series at time *t*, *σ*_*t*_ is predicted volatility, and *N*(*α*) is the quantile of the standard normal distribution that corresponds to the VaR probability. The Expected Shortfall (ES) equation is given as:

36 $$ES={\mu}_t+{\sigma}_t\frac{f\left(N\left(\alpha \right)\right)}{1-\alpha }$$

where *μ*_*t*_ and *σ*_*t*_ are defined as above and *f*(*N*(*α*)) is the density function of the *α*^*th*^ quantile of the standard normal distribution. For further discussion see, Hendricks (1996), Scaillet (2004), Alexander (2009), Hull (2012), Fissler and Ziegel (2016), Taylor (2019).

To test the accuracy and effectiveness of the VaR model, we use three appropriate tests, the Kupiec, Christoffersen and Dynamic Quantile (DQ) tests. The Kupiec (1995) unconditional coverage test is a likelihood ratio test (*LR*_*UC*_) designed to assess whether the theoretical VaR failure rate given by the confidence level is statistically consistent with the empirical failure rate and is given by:

37 $${LR}_{UC}=2\log {\left(1-{N}_0/{N}_1\right)}^{N_1-{N}_0}{\left({N}_0/{N}_1\right)}^{N_0}-2\log {\left(1-\phi \right)}^{N_1-{N}_0}{\phi}^{N_0}$$

where *p* = *E*(*N*_0_/*N*_1_) is the expected ratio of VaR violations obtained by dividing the number of violations *N*_0_ to forecasting sample size *N*_1_ and, *ϕ* is the prescribed VaR level (Tang and Shieh, 2006). The Kupiec test is asymptomatically distributed (~*X*^2^(1)) with one degree of freedom.

Although the Kupiec test is widely used, one of its disadvantages is that it only focuses on the number of violations, i.e., when the loss in the return of an asset exceeds the expected value of the VaR model. However, it is often observed that these violations occur in clusters. Clustering of violations (and hence, losses) is something that risk managers would ideally like to be able to determine. Thus, the conditional coverage test of Christoffersen (1998) is proposed to examine not only the frequency of VaR failures but also the time and duration between two violations. The model adopts a similar theoretical framework to Kupiec, with the extension of and additional statistic for the independence of exceptions, as such:

38 $${LR}_{CC}=2\log \Big({\left(1-{p}_{01}\right)}^{n_{00}}{p}_{01}^{n_{01}}\left(1-{p}_{11}\Big){}^{n_{10}}{p}_{11}^{n_{11}}\right)-2\log \left({\left(1-{p}_0\right)}^{n_{00}+{n}_{10}}{p_0}^{n_{01}+{n}_{11}}\right)$$

where *p*_*ij*_ is the expected ratio of violations on state *i*, while *j* occurs on the previous period, and *n*_*ij*_ is defined as the number of days *for* (*i*, *j* = 0, 1). For the detailed procedure and further information see; Christoffersen (1998), Jorion (2002), Campbell (2005), and Dowd (2006). In addition to the Kupiec and Christoffersen tests, we use the Dynamic Quantile (DQ) test proposed by Engle and Manganelli (2004). The DQ test is based on a linear regression model to measure whether the current violations are linked to the past violations. The authors define a demeaned process of violation as:

39 $${Hit}_t(a)={I}_t(a)-a=\left\{\begin{array}{c}1-a,\kern1em if\ {x}_t<{VaR}_t(a),\\ {}-a,\kern4em otherwise.\end{array}\right.$$

where *Hit*_*t*_(*a*) is the conditional expectation and equal to 1 − *a* if the observed return series is less than the VaR quantile, and −*a* otherwise. The sequence assumes that the conditional expectation of *Hit*_*t*_(*a*) must be zero at time *t* − 1 (see Giot and Laurent, 2004). The test statistic for the DQ is given as follows:

40 $$DQ=\frac{{\hat{\psi}}^{\prime }{Q}^{\prime }Q\hat{\psi}}{a\left(1-a\right)},$$

where *Q* denotes the matrix of explanatory variables and $\hat{\psi}$ indicates the OLS estimator. The proposed test statistic follows a chi-squared distribution ${X}_q^2$, in which *q* =  *rank* (*X*_*t*_).

## Data

In this research, ten Asian countries and their widely accepted indices are chosen. These markets are: the Nikkei 225 Index (NIKKEI) from Japan, the Hang Seng Index (HSI) from Hong Kong, the Korea Composite Stock Market Index (KOSPI) from South Korea, the Taiwan Capitalization Weighted Stock Index (TAIEX) from Taiwan, the Straits Times Index (STI) from Singapore, the SSE Composite Index (SSE) from China, the PSE Composite Index (PSE) from the Philippines, the Stock Exchange of Thailand Index (SET) from Thailand, the Kuala Lumpur Composite Index (KLCI) from Malaysia, and the Jakarta Stock Exchange Composite Index (JCI) from Indonesia.

The sample period spans from 12/09/1994 to 05/03/2018, with Table 1 reporting the selected markets (and indices) and sample sizes (including out-of-sample forecast period) for each market, respectively. This period is selected based on data availability and covers important financial events such as the Asian financial crisis of 1997-98 and the global financial crisis of 2008. Table 2 presents the key descriptive statistics for the total data sample for each index. The mean fluctuates between 0.0047 and 0.0448 for daily returns. Indonesia outperforms other markets while the Thai stock market performs worst. The return distribution is not symmetrical, with the series exhibiting skewness. The values in Table 2 suggest that half the selected markets exhibit negative skewness, with the other half indicating positive skewness.^2^ The results also suggest the presence of excess kurtosis, which indicate a larger number of extreme shocks (of either sign) than under a normal distribution. Of further note, China has the highest maximum value, while Singapore and Taiwan have the lowest maximum values. The greatest single-day increase is in China’s SSE of 26.99% and the largest decline occurs in Malaysia’s KLCI with -24.15%. Singapore’s STI and Taiwan’s TAIEX Indices have the smallest gap between daily minimum and maximum values of -8.70% and 7.53% and -6.98% and 6.52% respectively. This result indicates lower volatility compared to others, which is also seen in the standard deviation values.^3^

**Table 2:** Summary of descriptive statistics for daily return series

	NIKKEI	STRAITS TIMES INDEX	HANG SENG INDEX	KUALA LUMPUR COMPOSITE INDEX	JAKARTA COMPOSITE INDEX	SET INDEX	SSE INDEX	TAIEX	KOSPI	PSE INDEX
Mean	0.0294	0.0105	0.024194	0.012354	0.044841	0.004651	0.028739	0.015257	0.015445	0.015835
Median	0.030928	0.02846	0.0511	0.025455	0.090305	0.015914	0.065357	0.043451	0.050211	0.021669
Maximum	13.23458	7.531083	17.2471	20.81737	13.12768	11.34953	26.99277	6.52462	11.28435	16.1776
Minimum	-12.11103	-8.695982	-14.73468	-24.15339	-12.73214	-16.06325	-17.90509	-6.975741	-12.8047	-13.08869
Std. Dev.	1.504108	1.141326	1.60473	1.267938	1.52564	1.526721	1.761533	1.36745	1.66492	1.393054
Skewness	-0.300663	-0.266133	0.064089	0.502157	-0.19832	0.049086	0.195354	-0.182956	-0.291322	0.162169
Kurtosis	8.540723	8.37642	13.32528	65.37193	11.58383	10.95738	18.86232	5.815682	8.152126	14.21301
Probability	0.000	0.0000	0.0000	0.0000	0.0000	0.0000	0.0000	0.0000	0.0000	0.0000
Observations	5748	4689	5750	5740	5688	5703	5656	5892	5942	5728
Sample	12/09/1994 5/03/2018	8/31/1999 5/03/2018	1/10/1995 5/03/2018	1/10/1995 5/03/2018	1/11/1995 5/03/2018	1/11/1995 5/03/2018	1/10/1995 5/03/2018	1/10/1995 5/03/2018	1/10/1995 5/03/2018	1/10/1995 5/03/2018

## Empirical results

Table 3 presents the forecasting performance results for daily return series based on the Mean Absolute Error (MAE), Mean Absolute Percentage Error (MAPE), Root Mean Squared Error (RMSE), Quasi-Likelihood (QLIKE) and Mean Squared Error (MSE) measures. The out-of-sample forecasts are obtained using the ten ANN models and four benchmark models. The overall results suggest that the benchmark models provide superior forecasts based on the MAE criterion for seven of the ten indices, with the only exceptions of STI, KLCI and JCI indices. The result for the KLCI index is consistent with the study of Yao et al. (1999). According to the MAPE criterion, ANN models clearly outperform the benchmark models. Notably, the RNN, RBFN and PNN models provide the lowest MAPE values across multiple indices. In terms of the RMSE loss function, the EGARCH model achieves the best results in KLCI and TAIEX indices, whereas the GARCH model performs the worst among all. LSTM model tends to provide more accurate forecast results compared to other models. This contrasts with the work of Selvin et al. (2017), although supports the findings of Chen et al. (2015) and Nelson et al. (2017). The QLIKE and MSE error criteria find substantial support for the prediction power of ANN-based models with the only exception of STI, KLCI and TAIEX indices, for which they provide either mixed results or favour traditional forecasting models. The adaptive and coactive network-based hybrid models of ANFIS and CANFIS indicate the lowest prediction errors specifically in HANG SENG, TAIEX and PSE indices, which supports Chang et al. (2008), Boyacioglu and Avci (2010), and Kristjanpoller and Michell (2018). The comparative predictive performance of standard NN, neuro-fuzzy and deep learning models indicate robust results compared to conventional methods for more occasions than the reverse. More specifically, the LSTM provides superior forecasts for six of the ten markets based on the MSE criterion, which justifies its preferential role in long-term time series predictions given its memory cell properties (Kim and Kang, 2019). Other deep learning models, such as RNN, MLP and RBFN, are superior in three, three and four occasions respectively. In addition to the findings of Yap et al. (2021) on using deep learning models for predicting short-term movements and market trends in Asian tiger countries, the present results show that deep learning models are preferred in forecasting a wider range of markets. Furthermore, neuro-fuzzy models are favoured specifically for the NIKKEI, HANG SENG, SSE, TAIEX and PSE indices, despite it clearly underperforming for the remaining markets. Although Atsalakis et al. (2016) state that Neuro-fuzzy models are more preferred for turbulent times and shorter-term predictions given their rapid learning capabilities, these results show that neuro-fuzzy models also offer promising results over longer-term periods. GFN, MFN and PNN models indicate outperformance in seven, five and two occasions respectively. Notably, the MFN is clearly preferred for KLCI index where four out of five losses indicate preference. The GFN model reports its lowest errors based on RMSE, QLIKE and MSE for JCI index. The PNN model is the weakest among all ANN models where it is only preferable based on MAPE criterion for TAIEX and HANG SENG indices. This result supports the view of Chen et al. (2003) for TAIEX index where PNN also produces enhanced predictive power compared to parametric benchmark. However, as indicated by Wang and Wu (2017), the overall weaker performance of PNN might be due to its high computational complexity in the standard architecture which causes difficulties in the estimation of parameters.

**Table 3:** Comparison of forecast performance measures for daily return series

NIKKEI INDEX						HANG SENG INDEX					
Model	MAE	MAPE	RMSE	QLIKE	MSE	Model	MAE	MAPE	RMSE	QLIKE	MSE
LSTM	0.32	6.66	0.46	0.18	**0.21**	LSTM	0.29	7.10	0.44	**0.15**	**0.19**
RNN	0.31	5.69	0.47	0.22	0.22	RNN	0.30	5.44	0.46	NA	0.21
MLP	0.35	6.63	0.51	0.55	0.26	MLP	0.37	9.48	0.54	1.31	0.29
RBFN	0.32	6.56	0.48	0.21	0.23	RBFN	0.34	10.93	0.46	0.45	0.21
ANFIS	0.27	**5.43**	0.51	**0.11**	0.28	ANFIS	0.33	6.76	**0.39**	0.19	0.28
CANFIS	0.33	5.44	0.46	0.13	**0.21**	CANFIS	0.37	6.55	**0.38**	0.22	0.33
PNN	0.40	6.54	0.60	5.74	0.36	PNN	0.39	**5.42**	0.61	6.83	0.37
GFN	0.32	7.06	0.46	0.18	**0.21**	GFN	0.30	7.38	0.45	**0.15**	0.20
MFN	0.32	6.53	**0.45**	0.18	**0.21**	MFN	0.34	9.71	0.46	0.16	0.21
ANN Fc	0.33	6.28	0.49	0.83	0.24	ANN Fc	0.34	7.64	0.47	1.18	0.25
GARCH(1,1)	0.34	10.49	0.69	1.56	0.48	GARCH(1,1)	0.26	10.32	0.73	1.46	0.53
EGARCH(1,1)	**0.25**	10.56	0.69	1.54	0.47	EGARCH(1,1)	**0.25**	10.23	0.70	1.46	0.48
MACD	0.55	13.50	1.27	1.56	0.59	MACD	0.91	9.80	1.01	1.91	0.29
NAÏVE	0.41	6.59	0.73	5.71	0.34	NAIVE	0.42	7.81	0.56	6.89	0.38
STRAITS TIMES INDEX						SET INDEX					
Model	MAE	MAPE	RMSE	QLIKE	MSE	Model	MAE	MAPE	RMSE	QLIKE	MSE
LSTM	**0.19**	4.81	0.26	0.33	0.07	LSTM	0.37	0.60	0.46	**0.06**	0.21
RNN	**0.19**	4.82	0.26	0.32	0.07	RNN	0.24	0.80	**0.38**	0.17	**0.15**
MLP	0.23	6.66	0.28	**0.26**	0.08	MLP	0.31	**0.15**	0.43	0.84	0.18
RBFN	**0.19**	**4.34**	0.26	1.66	0.07	RBFN	0.25	0.80	**0.38**	0.55	**0.15**
ANFIS	0.44	4.57	0.35	0.44	0.13	ANFIS	0.24	0.33	0.54	0.47	0.19
CANFIS	0.29	5.33	0.28	0.52	0.11	CANFIS	0.27	0.28	0.57	0.41	0.22
PNN	0.24	4.90	0.35	3.65	0.13	PNN	0.34	4.51	0.51	3.34	0.26
GFN	0.21	6.12	0.27	0.29	0.07	GFN	0.27	0.60	**0.38**	0.07	**0.15**
MFN	0.20	5.32	0.26	0.31	0.07	MFN	0.26	0.61	**0.38**	0.08	**0.15**
ANN Fc	0.24	5.21	0.29	0.86	0.09	ANN Fc	0.28	0.96	0.45	0.67	0.18
GARCH(1,1)	0.91	9.68	0.20	0.50	**0.04**	GARCH(1,1)	**0.19**	10.53	0.67	1.21	0.45
EGARCH(1,1)	0.91	9.50	0.20	0.49	**0.04**	EGARCH(1,1)	**0.19**	10.51	0.67	1.15	0.45
MACD	0.80	10.20	0.44	1.94	0.26	MACD	0.55	9.80	0.67	2.03	0.67
NAIVE	0.30	5.94	**0.09**	4.77	0.19	NAIVE	0.39	4.50	0.47	3.55	0.34
KUALA LUMPUR COMPOSITE INDEX						JAKARTA COMPOSITE INDEX					
Model	MAE	MAPE	RMSE	QLIKE	MSE	Model	MAE	MAPE	RMSE	QLIKE	MSE
LSTM	0.18	5.77	0.23	0.54	**0.05**	LSTM	0.29	6.37	**0.40**	**0.01**	**0.16**
RNN	0.14	6.11	0.23	0.52	0.05	RNN	0.30	6.68	0.41	**0.01**	0.17
MLP	0.24	4.53	0.31	0.58	0.09	MLP	0.33	6.09	0.47	0.80	0.22
RBFN	0.21	6.28	0.28	0.32	0.08	RBFN	**0.27**	4.35	**0.40**	1.06	**0.16**
ANFIS	0.17	3.33	0.29	0.42	0.09	ANFIS	0.37	7.43	0.57	0.26	0.24
CANFIS	0.18	3.89	0.37	0.45	0.10	CANFIS	0.48	8.55	0.41	0.18	0.23
PNN	0.19	4.04	0.29	2.39	0.09	PNN	0.35	3.95	0.54	7.65	0.29
GFN	0.16	6.07	0.23	1.21	**0.05**	GFN	0.28	6.23	**0.40**	**0.01**	**0.16**
MFN	**0.15**	**1.42**	**0.22**	0.84	**0.05**	MFN	0.29	6.60	0.41	0.02	0.17
ANN Fc	0.18	4.60	0.27	0.81	0.07	ANN Fc	0.33	6.25	0.45	1.11	0.20
GARCH(1,1)	0.68	10.00	0.23	0.06	**0.05**	GARCH(1,1)	0.29	10.94	0.42	0.72	0.24
EGARCH(1,1)	0.67	10.05	**0.22**	**0.05**	**0.05**	EGARCH(1,1)	0.29	10.99	0.42	0.76	0.23
MACD	0.44	10.21	0.57	1.92	2.40	MACD	0.37	10.75	0.93	2.03	6.62
NAIVE	0.27	4.42	0.66	3.04	0.22	NAIVE	0.38	**3.59**	0.80	2.64	0.35
SSE INDEX						TAIEX INDEX					
Model	MAE	MAPE	RMSE	QLIKE	MSE	Model	MAE	MAPE	RMSE	QLIKE	MSE
LSTM	0.40	6.50	0.53	0.29	0.28	LSTM	0.25	4.89	0.34	0.11	0.12
RNN	0.40	6.44	0.52	0.27	0.27	RNN	0.26	5.17	0.35	0.08	0.12
MLP	0.40	4.90	0.58	0.31	0.34	MLP	0.30	6.44	0.38	**0.01**	0.15
RBFN	0.34	4.29	0.51	0.33	**0.26**	RBFN	0.25	4.52	0.35	0.54	0.12
ANFIS	0.37	8.43	0.49	**0.25**	0.29	ANFIS	0.36	4.77	**0.31**	0.29	0.12
CANFIS	0.36	7.56	0.57	0.28	**0.26**	CANFIS	0.48	6.49	0.37	0.43	**0.10**
PNN	0.45	4.90	0.66	5.36	0.43	PNN	0.33	**4.34**	0.47	8.15	0.22
GFN	0.34	4.04	0.51	0.30	**0.26**	GFN	0.26	5.37	0.35	0.09	0.12
MFN	0.38	5.91	0.52	**0.25**	0.27	MFN	0.26	5.53	0.35	0.08	0.12
ANN Fc	0.38	5.89	0.54	0.85	0.30	ANN Fc	0.31	5.28	0.36	1.09	0.13
GARCH(1,1)	0.34	10.47	0.69	1.70	0.48	GARCH(1,1)	**0.16**	9.98	0.32	1.03	**0.10**
EGARCH(1,1)	**0.33**	10.52	0.69	1.69	0.48	EGARCH(1,1)	**0.16**	9.84	**0.31**	1.02	**0.10**
MACD	1.12	**1.29**	0.97	1.74	1.33	MACD	0.64	10.25	0.68	1.75	0.17
NAIVE	0.47	7.51	**0.36**	6.64	0.52	NAIVE	0.39	5.89	0.71	4.09	0.30
KOSPI INDEX						PSE INDEX					
Model	MAE	MAPE	RMSE	QLIKE	MSE	Model	MAE	MAPE	RMSE	QLIKE	MSE
LSTM	0.24	9.12	0.36	**0.03**	**0.13**	LSTM	0.25	9.53	0.37	**0.01**	**0.13**
RNN	0.28	5.97	0.38	0.07	0.14	RNN	0.27	10.27	0.38	0.03	0.14
MLP	0.27	12.97	0.40	1.23	0.16	MLP	0.27	9.80	0.40	0.07	0.16
RBFN	0.39	6.14	0.46	0.07	0.21	RBFN	0.27	5.44	0.41	0.03	0.17
ANFIS	0.76	9.76	0.56	0.28	0.31	ANFIS	**0.18**	5.54	0.18	0.07	0.19
CANFIS	0.63	10.19	0.74	0.44	0.34	CANFIS	0.19	5.61	**0.12**	0.09	**0.13**
PNN	0.34	7.23	0.50	0.14	0.25	PNN	0.34	8.41	0.50	0.06	0.25
GFN	0.26	6.24	0.37	0.09	0.14	GFN	0.26	10.11	0.37	**0.01**	0.14
MFN	0.25	6.49	0.36	0.10	**0.13**	MFN	0.28	11.81	0.38	0.03	0.15
ANN Fc	0.38	8.23	0.46	0.27	0.20	ANN Fc	0.26	8.50	0.35	0.04	0.16
GARCH(1,1)	**0.18**	10.06	0.49	1.01	0.24	GARCH(1,1)	**0.18**	9.82	0.51	1.24	0.26
EGARCH(1,1)	**0.18**	10.09	0.48	1.00	0.23	EGARCH(1,1)	**0.18**	9.73	0.50	1.22	0.25
MACD	0.73	10.20	1.44	1.70	0.24	MACD	0.41	10.42	0.88	1.63	0.24
NAIVE	0.45	**4.62**	**0.35**	3.71	0.43	NAIVE	0.36	**5.42**	**0.12**	4.47	0.30

Bolded text indicates the preferred model according to each forecast evaluation measure

To provide some further understanding of the nature of the results, we consider the cumulative MSE and QLIKE plots for the ANN and GARCH models. The cumulative plots allow us to consider whether any forecast improvement occurs consistently over the sample period or whether it is associated with a particular date or event. To summarise the information across the nine different ANN models, we use the combined forecast series as defined by equation (23), while the same approach is undertaken to obtain a combined GARCH and EGARCH forecast.

**Fig. 1.**
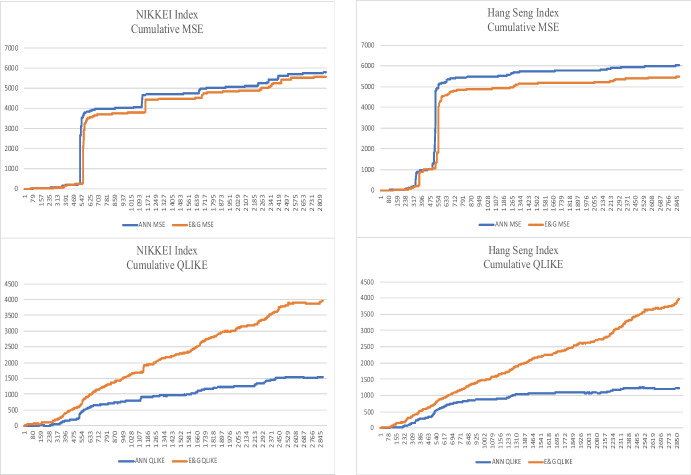

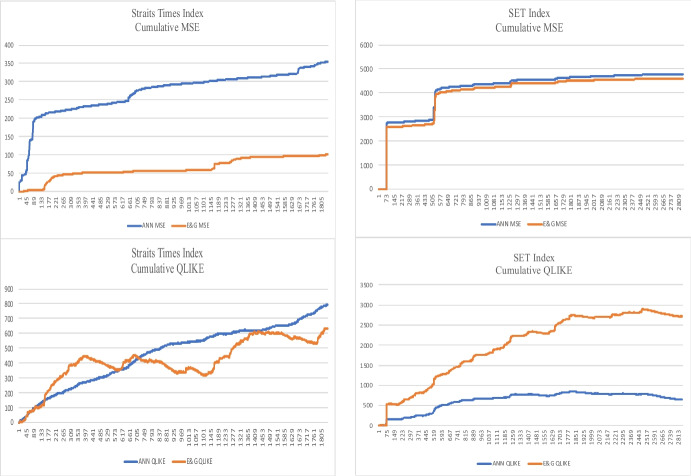

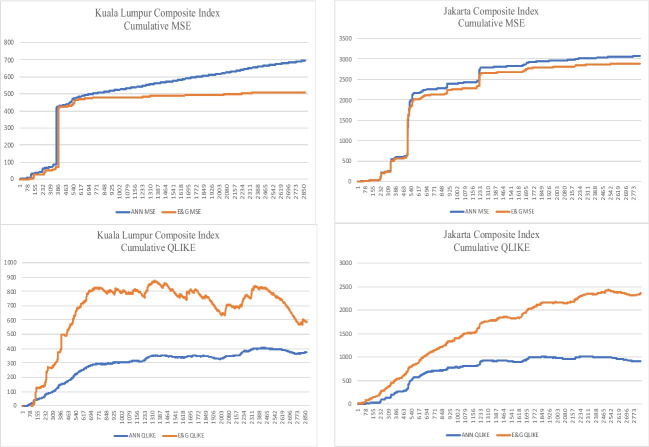

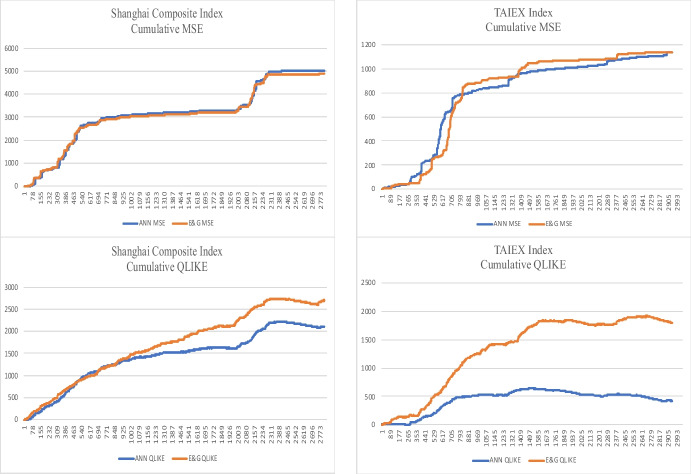

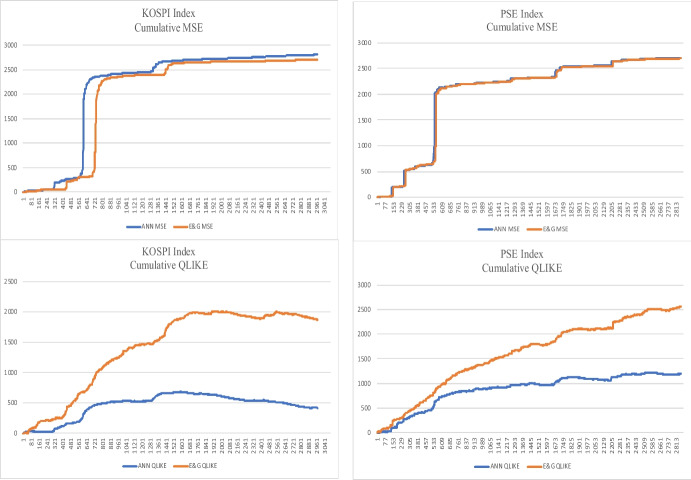
Comparison of Cumulative Forecasting Performance. **Notes:** ANN indicates the combination of ANN-based models, while E&G shows the combination of EGARCH and GARCH models.

Figure 1 presents the comparison of the cumulative MSE and QLIKE error functions over the out-of-sample period for the combined ANN and GARCH models for each index. One clear characteristic across the graphs is the jump associated with the 2007-2008 crisis in almost all markets and which is reflected in the MSE loss function more noticeably than the QLIKE error criterion. This also clearly highlights that large volatility increases that occur during turbulent times present difficulties in forecasting and applies to both ANN models and GARCH models. To consider a further example of the same effect, we can observe a jump in the Shanghai Composite Index during the 2015-2016 Chinese Stock Market turbulence.

In considering the relative performance of ANN versus GARCH models, we can see that for the cumulative MSE graphs, the two forecast series track each other closely with GARCH approach typically offering a smaller value. One notable exception is for the STI where the GARCH model is clearly preferred throughout. Moreover, the forecast improvement with the GARCH model largely arises after the financial crisis period, with this being most obvious for the KLCI. Considering the cumulative forecasts for the QLIKE measure, we can see that the ANN approach consistently outperforms the GARCH model, with the except of the STI. Moreover, this forecast improvement is not associated with a particular event, as we see with the MSE but over the whole forecast sample the ANN model shows an improving outperformance i.e., the gap between the two cumulative series expands. Furthermore, for most of the series, we see the ANN QLIKE function exhibiting greater stability compared to the GARCH model where there is a continuous increase in QLIKE values.

Following the studies of Hansen et al. (2011), Wang et al. (2016), and Liu et al. (2017), we also consider Model Confidence Set (MCS) test with a confidence level of 75% which allows us to compare the given model set in MCS framework with a *p*-value larger than 0.25. Table 4 exhibits the MCS test for both MSE and QLIKE metrics based on the out-of-sample forecasting results. The bold values in the table denotes the optimal model chosen by MCS, while the test also considers the number of other models with EPA at the given confidence level. The corresponding results of the MCS test indicate the ANN class of models are significantly better than the benchmark models. Specifically, LSTM model is preferred on five occasions based on the MSE criterion and three occasions based on the QLIKE loss function. Moreover, the QLIKE loss function supports the superiority of MFN model in five markets, while traditional methods are eliminated in most cases. In summary, the MCS test results confirm the superiority of ANN models over the benchmark models of GARCH, EGARCH, MACD and Naïve models.

**Table 4:** The Model Confidence Set test results for individual forecasts given the MSE and QLIKE loss functions

	NIKKEI INDEX	HANG SENG INDEX	STRAITS TIMES INDEX	SET INDEX	KUALA LUMPUR COMPOSITE INDEX	JAKARTA COMPOSITE INDEX	SSE INDEX	TAIEX INDEX	KOSPI INDEX	PSE INDEX
					**MSE**					
LSTM	0.9480	**1.0000**	**1.0000**	0.0010	**1.0000**	0.0000	0.0488	0.1366	**1.0000**	**1.0000**
RNN	**1.0000**	0.1180	0.0000	0.3564	0.0000	0.0000	0.0000	0.0000	0.0368	0.0000
MLP	0.4280	0.0000	0.0000	0.0000	0.0000	0.0000	0.0000	0.0000	0.0000	0.0000
RBFN	0.0000	0.0000	0.0000	0.0000	0.0000	0.0000	0.0000	0.0000	0.0636	0.0000
ANFIS	0.0000	0.7749	0.0000	0.0000	0.0000	**1.0000**	0.0000	0.9654	0.0000	0.0000
CANFIS	0.6689	0.0000	0.0000	0.0000	0.8959	0.0000	0.0000	0.0000	0.0000	0.0443
PNN	0.0184	0.0798	0.0000	0.2074	0.0000	0.0000	0.0000	**1.0000**	0.0000	0.0000
GFN	0.9990	0.3014	0.0000	**1.0000**	0.0000	0.0000	**1.0000**	0.8248	0.0032	0.0000
MFN	0.5490	0.0000	0.0114	0.0000	0.0000	0.0000	0.0000	0.5430	0.0000	0.0000
ANN Fc	0.0000	0.0000	0.0000	0.0000	0.0000	0.0000	0.0000	0.0000	0.0000	0.0000
GARCH(1,1)	0.2872	0.0000	0.0000	0.0000	0.0000	0.0000	0.3966	0.0000	0.5450	0.0000
EGARCH(1,1)	0.4280	0.0000	0.9887	0.0000	0.0000	0.0000	0.2432	0.0000	0.9458	0.0000
MACD	0.0000	0.0000	0.0000	0.0000	0.0000	0.0000	0.0000	0.0000	0.0000	0.0000
NAIVE	0.0000	0.0000	0.0000	0.0000	0.0000	0.0000	0.0000	0.0000	0.0000	0.0000
					**QLIKE**					
LSTM	0.0000	0.0000	**1.0000**	0.0000	0.0000	0.0000	**1.0000**	**1.0000**	0.0000	0.0000
RNN	0.0000	0.0000	0.7320	0.0000	0.0000	0.0000	0.0000	0.0000	0.4932	0.0000
MLP	0.8800	0.0000	0.0000	0.0000	0.3349	0.0000	0.0000	0.0000	0.0000	0.0000
RBFN	0.0000	0.0000	0.0000	0.0000	0.0000	0.0000	0.0000	0.0000	0.0000	0.0000
ANFIS	0.7339	0.0000	0.0000	0.0000	0.0000	0.2957	0.0000	0.0000	0.0000	0.5639
CANFIS	0.0000	0.0000	0.2740	0.0000	0.0000	0.0000	0.0000	0.3354	0.0000	0.0000
PNN	0.0000	0.0000	0.0000	0.0000	0.0000	**1.0000**	0.0000	0.0000	0.0000	0.0000
GFN	0.0000	0.0000	0.0000	0.0000	**1.0000**	0.0000	0.0000	0.0000	0.0000	0.0000
MFN	**1.0000**	**1.0000**	0.0000	**1.0000**	0.0000	0.0000	0.0000	0.0000	**1.0000**	**1.0000**
ANN Fc	0.0000	0.0000	0.0000	0.0000	0.0000	0.0000	0.0000	0.0000	0.0000	0.0000
GARCH(1,1)	0.0000	0.0000	0.0000	0.0000	0.0000	0.0000	0.5530	0.0000	0.0000	0.0000
EGARCH(1,1)	0.0000	0.2239	0.0000	0.0000	0.0000	0.0000	0.0000	0.0000	0.0000	0.0000
MACD	0.0000	0.0000	0.0000	0.0000	0.0000	0.0000	0.0000	0.0000	0.0000	0.0000
NAIVE	0.0000	0.0000	0.0000	0.0000	0.0000	0.0000	0.0000	0.0000	0.0000	0.0000

The table reports the MCS test on both MSE and QLIKE loss functions. The confidence level is set at *α*= 0.2. The forecasting models with Equal Predictive Ability (EPA) at 75% level is bolded in the table

Table 5 presents the daily VaR and Expected Shortfall statistics as well as the corresponding test results. Examining Table 5, the lowest average VaR failure rate at the 1% level is mainly achieved by the hybrid models of ANFIS and CANFIS, while the benchmark models of GARCH and EGARCH report lowest values in KLCI and SET indices. The PNN model provides the preferred average failure rate for KOSPI index, while the RBFN and PNN models are preferred for the SSE index. In contrast, the LSTM, RNN and MLP models fail to provide minimum VaR rates for any of the selected indices and for which they tend to underestimate potential risks. As recently proposed by Basel Committee in 2017, there is a move regarding quantitative risk measures from VaR to ES (Expected Shortfall). In forecasting ES, the MLP model is preferred at 1% and 5% levels for the SSE, PSE, STI and HANG SENG indices. Furthermore, the RBFN, MFN and PNN models are preferred in both confidence levels for NIKKEI, KLCI and KOSPI indices. Accordingly, it can be inferred that the ANN models are the most suitable across all competing models in terms of Expected Shortfall at all selected confidence levels. The accuracy and reliability of the VaR forecasts are also tested as proposed by Basel Ι and Basel ΙΙ. Based on the tests of Kupiec, Christoffersen and DQ, the results report that none of the models reject the null hypothesis of expected VaR violation (Kupiec’s unconditional coverage test), the independence exceptions of VaR (Christoffersen’s conditional coverage test), and violations of VaR occurred correlated (Dynamic Quantile).

**Table 5:** Summary of Risk Management analysis and Backtesting results for daily return series

NIKKEI INDEX							HANG SENG INDEX						
	Avg.FR (1%)	Sig. LRcc	Sig. LRuc	Sig. DQ Test	ES (1%)	ES (5%)		Avg.FR (1%)	Sig. LRcc	Sig. LRuc	Sig. DQ Test	ES (1%)	ES (5%)
LSTM	0.0289	ALL	ALL	ALL	0.2503	0.2660	LSTM	0.0290	ALL	ALL	ALL	0.1619	0.1899
RNN	0.0287	ALL	ALL	ALL	0.1923	0.2216	RNN	0.0290	ALL	ALL	ALL	0.1085	0.1463
MLP	0.0290	ALL	ALL	ALL	0.0266	0.1105	MLP	0.0303	ALL	ALL	ALL	**-0.5117**	**-0.2608**
RBFN	0.0271	ALL	ALL	ALL	-**0.0367**	**0.0058**	RBFN	0.0288	ALL	ALL	ALL	0.0511	0.1019
ANFIS	0.0211	ALL	ALL	ALL	0.0313	0.0424	ANFIS	**0.0254**	ALL	ALL	ALL	0.0448	0.0822
CANFIS	**0.0124**	ALL	ALL	ALL	0.0114	0.0193	CANFIS	0.0258	ALL	ALL	ALL	0.0535	0.0998
PNN	0.0271	ALL	ALL	ALL	**-0.0367**	0.0058	PNN	0.0288	ALL	ALL	ALL	0.0511	0.1019
GFN	0.0290	ALL	ALL	ALL	0.2278	0.2594	GFN	0.0294	ALL	ALL	ALL	0.1444	0.1817
MFN	0.0308	ALL	ALL	ALL	0.2748	0.3143	MFN	0.0308	ALL	ALL	ALL	0.2386	0.2724
GARCH(1,1)	0.0269	ALL	ALL	ALL	0.0916	0.0983	GARCH(1,1)	0.0313	ALL	ALL	ALL	0.0568	0.0634
EGARCH(1,1)	0.0262	ALL	ALL	ALL	0.0783	0.0900	EGARCH(1,1)	0.0314	ALL	ALL	ALL	0.0241	0.0293
STRAITS TIMES INDEX							SET INDEX						
	Avg.FR (1%)	Sig. LRcc	Sig. LRuc	Sig. DQ Test	ES (1%)	ES (5%)		Avg.FR (1%)	Sig. LRcc	Sig. LRuc	Sig. DQ Test	ES (1%)	ES (5%)
LSTM	0.0277	ALL	ALL	ALL	0.2526	0.2618	LSTM	0.0291	ALL	ALL	ALL	0.3235	0.3341
RNN	0.0279	ALL	ALL	ALL	0.1608	0.1854	RNN	0.0293	ALL	ALL	ALL	0.1908	0.2216
MLP	0.0265	ALL	ALL	ALL	**-0.0213**	**0.0269**	MLP	0.0301	ALL	ALL	ALL	**-0.1459**	0.0803
RBFN	0.0276	ALL	ALL	ALL	0.1051	0.1358	RBFN	0.0291	ALL	ALL	ALL	0.1702	0.2003
ANFIS	0.0277	ALL	ALL	ALL	0.1148	0.1225	ANFIS	**0.0258**	ALL	ALL	ALL	0.1445	0.1839
CANFIS	0.0270	ALL	ALL	ALL	0.1053	0.1090	CANFIS	0.0270	ALL	ALL	ALL	0.1735	0.2998
PNN	0.0276	ALL	ALL	ALL	0.1051	0.1358	PNN	0.0291	ALL	ALL	ALL	0.1702	0.2003
GFN	0.0270	ALL	ALL	ALL	0.1467	0.1660	GFN	0.0284	ALL	ALL	ALL	0.2142	0.2328
MFN	0.0272	ALL	ALL	ALL	0.1078	0.1360	MFN	0.0297	ALL	ALL	ALL	0.2528	0.2770
GARCH(1,1)	**0.0241**	ALL	ALL	ALL	0.0352	0.0384	GARCH(1,1)	**0.0258**	ALL	ALL	ALL	0.0698	**0.0753**
EGARCH(1,1)	0.0242	ALL	ALL	ALL	0.0313	0.0349	EGARCH(1,1)	**0.0258**	ALL	ALL	ALL	0.0669	0.0759
KUALA LUMPUR COMPOSITE INDEX							JAKARTA COMPOSITE INDEX						
	Avg.FR (1%)	Sig. LRcc	Sig. LRuc	Sig. DQ Test	ES (1%)	ES (5%)		Avg.FR (1%)	Sig. LRcc	Sig. LRuc	Sig. DQ Test	ES (1%)	ES (5%)
LSTM	0.0256	ALL	ALL	ALL	0.0534	0.0668	LSTM	0.0282	ALL	ALL	ALL	0.2712	0.2811
RNN	0.0256	ALL	ALL	ALL	-0.0282	0.0091	RNN	0.0287	ALL	ALL	ALL	0.1276	0.1646
MLP	0.0288	ALL	ALL	ALL	0.0423	0.1834	MLP	0.0288	ALL	ALL	ALL	**-0.0162**	0.1190
RBFN	0.0270	ALL	ALL	ALL	0.0321	0.0880	RBFN	0.0297	ALL	ALL	ALL	0.1536	0.2131
ANFIS	0.0263	ALL	ALL	ALL	0.0422	0.0624	ANFIS	0.0255	ALL	ALL	ALL	0.2213	0.2464
CANFIS	0.0275	ALL	ALL	ALL	0.0375	0.0524	CANFIS	**0.0249**	ALL	ALL	ALL	0.1745	0.1930
PNN	0.0270	ALL	ALL	ALL	0.0321	0.0880	PNN	0.0297	ALL	ALL	ALL	0.1536	0.2131
GFN	0.0280	ALL	ALL	ALL	0.2437	0.2574	GFN	0.0289	ALL	ALL	ALL	0.1979	0.2298
MFN	0.0246	ALL	ALL	ALL	**-0.1234**	**-0.0736**	MFN	0.0286	ALL	ALL	ALL	0.2169	0.2427
GARCH(1,1)	**0.0241**	ALL	ALL	ALL	0.0255	0.0280	GARCH(1,1)	0.0266	ALL	ALL	ALL	0.0687	0.0735
EGARCH(1,1)	**0.0241**	ALL	ALL	ALL	0.0205	0.0243	EGARCH(1,1)	0.0265	ALL	ALL	ALL	0.0503	**0.0595**
SSE INDEX							TAIEX INDEX						
	Avg.FR (1%)	Sig. LRcc	Sig. LRuc	Sig. DQ Test	ES (1%)	ES (5%)		Avg.FR (1%)	Sig. LRcc	Sig. LRuc	Sig. DQ Test	ES (1%)	ES (5%)
LSTM	0.0320	ALL	ALL	ALL	0.5068	0.5175	LSTM	0.0287	ALL	ALL	ALL	0.2679	0.2827
RNN	0.0280	ALL	ALL	ALL	0.0475	0.0866	RNN	0.0286	ALL	ALL	ALL	0.2473	0.2686
MLP	0.0295	ALL	ALL	ALL	**-0.5657**	**-0.3355**	MLP	0.0283	ALL	ALL	ALL	0.0608	0.1483
RBFN	**0.0264**	ALL	ALL	ALL	-0.1772	-0.1306	RBFN	0.0278	ALL	ALL	ALL	**0.0484**	0.0895
ANFIS	0.0284	ALL	ALL	ALL	0.1945	0.2675	ANFIS	**0.0228**	ALL	ALL	ALL	0.1124	0.1639
CANFIS	0.0293	ALL	ALL	ALL	0.1424	0.1505	CANFIS	0.0247	ALL	ALL	ALL	0.1336	0.1469
PNN	**0.0264**	ALL	ALL	ALL	-0.1772	-0.1306	PNN	0.0278	ALL	ALL	ALL	**0.0484**	0.0895
GFN	0.0286	ALL	ALL	ALL	0.1222	0.1504	GFN	0.0285	ALL	ALL	ALL	0.1938	0.2184
MFN	0.0275	ALL	ALL	ALL	-0.0416	0.0011	MFN	0.0276	ALL	ALL	ALL	0.1582	0.1846
GARCH(1,1)	0.0282	ALL	ALL	ALL	0.0875	0.0930	GARCH(1,1)	0.0254	ALL	ALL	ALL	0.0927	0.0980
EGARCH(1,1)	0.0286	ALL	ALL	ALL	0.0817	0.0924	EGARCH(1,1)	0.0253	ALL	ALL	ALL	0.0770	**0.0846**
KOSPI INDEX							PSE INDEX						
	Avg.FR (1%)	Sig. LRcc	Sig. LRuc	Sig. DQ Test	ES (1%)	ES (5%)		Avg.FR (1%)	Sig. LRcc	Sig. LRuc	Sig. DQ Test	ES (1%)	ES (5%)
LSTM	0.0283	ALL	ALL	ALL	0.1768	0.1915	LSTM	0.0284	ALL	ALL	ALL	0.2154	0.2363
RNN	0.0282	ALL	ALL	ALL	0.2137	0.2343	RNN	0.0298	ALL	ALL	ALL	0.2344	0.2892
MLP	0.0288	ALL	ALL	ALL	-0.0790	0.0801	MLP	0.0296	ALL	ALL	ALL	**-0.3932**	**-0.1490**
RBFN	0.0280	ALL	ALL	ALL	0.1519	0.1794	RBFN	0.0254	ALL	ALL	ALL	-0.1084	-0.0704
ANFIS	0.0274	ALL	ALL	ALL	0.0327	0.0744	ANFIS	0.0249	ALL	ALL	ALL	-0.0233	-0.0361
CANFIS	0.0269	ALL	ALL	ALL	0.0459	0.0844	CANFIS	**0.0244**	ALL	ALL	ALL	-0.0124	-0.0487
PNN	**0.0150**	ALL	ALL	ALL	**-1.9910**	**-1.9910**	PNN	0.0254	ALL	ALL	ALL	-0.1084	-0.0704
GFN	0.0292	ALL	ALL	ALL	0.1974	0.2260	GFN	0.0270	ALL	ALL	ALL	0.0539	0.1022
MFN	0.0298	ALL	ALL	ALL	0.2767	0.2995	MFN	0.0301	ALL	ALL	ALL	0.3178	0.3458
GARCH(1,1)	0.0261	ALL	ALL	ALL	0.0772	0.0825	GARCH(1,1)	0.0271	ALL	ALL	ALL	0.0653	0.0722
EGARCH(1,1)	0.0260	ALL	ALL	ALL	0.0683	0.0807	EGARCH(1,1)	0.0259	ALL	ALL	ALL	0.0440	0.0580

Avg.FR indicates the failure rate of VaR at 1% significance level. LRcc and LRuc show the significance of the conditional (Christoffersen) and

unconditional (Kupiec) coverage tests at 1% level of significance, respectively. Sig. DQ Test denotes the significance of the Dynamic Quantile and ES

shows the Expected Shortfall at 1% and 5% confidence levels for the selected index

Bolded text indicates the preferred model for each measure

Overall, the results highlight the accuracy of the ANN class of models for volatility forecasting both in terms of statistical measures and economic, VaR and ES, metrics across a range of Asian stock markets. Notably, while there are exceptions, the results, similar to Zhang et al. (1998) and Cao and Wang (2020), suggests that the class of ANN models outperforms traditional forecasting methods across statistical and economic measures.

## Summary and conclusion

Volatility forecasting is essential for both practitioners and policymakers to enable them improve decision making and portfolio building, especially during periods of financial turbulence. This paper evaluates different Machine Learning methods in forecasting the volatility of ten Asian stock market indices, with the results compared against benchmark models. The empirical results for ANN models are promising. Out-of-sample forecast evaluation shows that ANN models are preferred for each index compared to the GARCH and EGARCH models. Notably, the results show that neural network prediction models exhibit improved forecasting accuracy across both statistical and economic-based metrics and offer new insights for market participants, academics, and policymakers. Although, it should be noted that the GARCH models do perform well across some individual series.

The contribution of this paper to the field of empirical finance is three-fold. First and foremost, this study explores key relevant machine learning models to address the problem of financial volatility forecasting. Previous studies tend to evaluate small sets of neural network methods. Using a wider range of ANN architectures has various advantages. For example, in stock market prediction exercises, recurrent ANNs are recommended due to their memory component features that increase prediction accuracy. Second, comprehensive performance measures for model evaluation are utilized, namely, both a range of statistical measures (RMSE, MAE, MAPE, MSE, QLIKE, and MCS) and economic-based ones (VaR and ES). Third, a wide range of Asian markets were studied in order to have an in-depth examination of an extended set of volatility models across markets that are less studied.

To extend the study, additional research could explore a further diverse set of ANN architectures. For example, according to Partaourides and Chatzis (2017), further regularization methods may increase the capacity of the machine learning systems. Moreover, hidden layers can be extended beyond two, more data frequencies can be added, and alternative input variables and activation functions can be studied. The value of such novel developments remains to be examined in future research endeavors.

## Data Availability

The data that support the findings of this study are available from Refinitiv Eikon, but restrictions apply to the availability of these data, which were used under license for the current study, and so are not publicly available. Data are however available from Refinitiv Eikon.

## Footnotes

1 According to Schaede (1991), the total estimated worldwide loss was US$1.71 trillion.

2 Eastman and Lucey (2008) suggest that in the event of negative skewness, most returns will be higher than average return, therefore market participants would prefer to invest in negatively skewed equities.

3 The Jarque-Bera statistic is significant at the 1% level for all series. Unit root rests support stationarity for returns.
